# Insulinoma Presenting with Long-Standing Depression, Primary Hypogonadism, and Sertoli Cell Only Syndrome

**DOI:** 10.1155/2013/926385

**Published:** 2013-12-22

**Authors:** Usman H. Malabu, Durgesh Gowda, Yong Mong Tan

**Affiliations:** Department of Endocrinology and Diabetes, James Cook University & Townsville Hospital, 100 Angus Smith Drive, Douglas, QLD 4814, Australia

## Abstract

The aim was to report an unusual case of insulinoma presenting with long-standing depression and primary testicular failure. We describe a 34-year-old male with clinical, laboratory, and radiologic data consistent with islet cell tumor and seminiferous tubule failure primary hypogonadism. The literature is reviewed relative to the component of this syndrome, and a possible association is discussed. The subject was investigated for a long-standing history of depression requiring medical attention because of mental confusion and slurred speech and was found to have an insulinoma. He was diagnosed with primary gonadal failure and physical examination showed no evidence of dysmorphic features. Chromosomal analysis revealed normal 46 XY and testicular biopsy showed Sertoli cell only syndrome (SCOS). Biochemistry revealed endogenous hyperinsulinism and histology confirmed an islet cell tumor. He remained euglycemic postoperatively and on followup. From this report, we emphasize drawing clinicians' attention to the possibility of an association between insulinoma and primary testicular failure and suggest consideration of this diagnosis in patients with hypergonadotropic hypogonadism who may present with infertility.

## 1. Introduction

Primary infertility and insulinoma have not been elucidated previously. Here, we report a patient with a history of long-standing symptoms of depression and hypogonadism and neuroglycopenic symptoms of recurrent episodic hypoglycemia. At presentation he complained of slurred speech, double vision, and feeling of blackout. Further assessment also revealed reduced libido and erectile dysfunction. Laboratory evaluation showed elevated serum FSH, azoospermia, and fasting hyperinsulinism confirmed to be an islet cell tumor. Hypoglycemic symptoms resolved following partial pancreatectomy.

## 2. Case Report

A 34-year-old male was referred for further assessment of insulinoma in 2011. Prior to that he had several years of depression including suicidal thoughts and had been seeing a clinical psychologist. His symptoms were that of episodic slurred speech, double vision, and blackout. The symptoms were worsened by exertion as evidenced by several home blood glucose readings of ≤2 mmol/L. He was never sexually active and did not smoke, drink alcohol, or use any recreational drugs or hypoglycemic medications. There was no family history of diabetes or hypoglycemia. Clinical examination revealed body weight to be 65 kg, height 174.6 cm, and body mass index 21.4 kg/m^2^. No evidence of disproportionate body segments was found. He had normal body and male pattern pubic hair and no gynecomastia. Both testes were of small volume measuring 8 mL and normal consistency. His sense of smell was unremarkable. Neck examination showed no evidence of neck mass or goiter and was clinically euthyroid.

**Figure 1:**
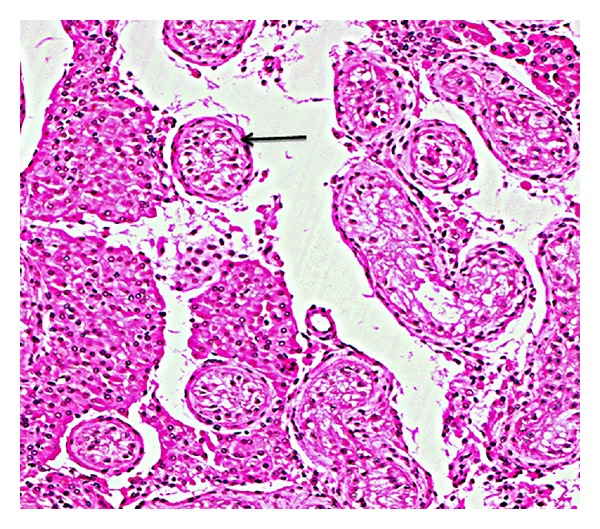
Histology of testicular biopsy of the patient showing seminiferous tubules containing Sertoli cells only (arrowed) with surrounding thickened tunica propria consistent with Sertoli cell only syndrome. Haematoxylin and eosin stain, magnification ×40.

The 72-hr fasting test was terminated prematurely owing to symptoms of hypoglycemia. The laboratory investigation was in keeping with endogenous hyperinsulinism. Notably, the serum glucose levels were low variously ranging between 38 and 55 mg/dL (normal range 70–110), with inappropriately normal serum insulin and C-peptide of 8–12 mU/L (fasting normal range 5–20) and 0.6—1.0 nmol/L (fasting normal range 0.5–1.0), respectively. Serum proinsulin was markedly elevated at 68.4 pmol/L (normal <13.3) and as expected beta-hydroxybutyrate concentration was consistently suppressed 0.10 mmol/L (<0.30). Serum and urine sulphonylurea screens were negative. Intraoperative pancreatic ultrasound revealed 7 × 11 mm adenoma localized on the head of the pancreas; however PET gallium octreotide scan was unable to detect any focal somatostatin receptor avid lesion. Histology of the surgical excision confirmed islet cell tumor. His other biochemistry was in keeping with primary testicular failure with elevated FSH and normal LH and low-normal serum testosterone (Table 1). Semen analysis revealed azoospermia. Karyotype showed 46 XY and no evidence of microdeletion on the Y chromosome. Testicular biopsy was consistent with Sertoli cell only syndrome (SCOS) with an occasional hyalinised tubule (Figure 1).

## 3. Discussion

We report the first case of insulinoma presenting with hypergonadotropic hypogonadism. The etiology of the nonobstructive azoospermia associated hypogonadism in our subject was investigated including chromosomal analysis which showed a normal male 46 XY pattern and testicular biopsy confirmed Sertoli cell only syndrome in line with the elevated serum FSH, normal or high LH, normal or low testosterone, and low inhibin B [1]. In determining the etiology of the SCOS there was no history of usage of any toxins, radiation exposure, and viral orchitis or Y microdeletion. We opined that the shrunken testes at presentation might suggest occurrence of testicular disease's at peri- or post pubertal stage in line with Jahnukainen et al. observation [2].

The association between the primary hypogonadism and insulinoma in our subject is not clear. Although coincidental finding is possible in that cause and effect had not been proven, we believe testicular injury from recurrent possibly long standing hypoglycemic attacks could be responsible. This phenomenon has not been reported in humans yet though testicular feminization was described in one case of insulinoma [3]. In support of our hypothesis, studies on animal model have confirmed testicular infarction caused by acute hypoglycemia [4, 5], but as of yet no link was reported with SCOS. Interestingly Van Demark and colleagues reported seminiferous tubular cell degeneration in adult but not in prepubertal rodents exposed to hypoglycemic coma [6]. The histologic pattern of SCOS in this report was consistent with germinal cell aplasia described in animal models observed in experimental studies involving severe hypoglycemia. SCOS was reported in humans to be caused by hypoxia [7], yet as far as we know there has been no link with hypoglycemia. With the reported high prevalence of testicular cancer in SCOS, our subject who had an islet cell tumour treated would need close surveillance for potential germinal cell tumors [8].

The implication of this report for diabetic subjects with recurrent hypoglycemia is obvious. It has long being established that testicular dysfunction, impotence, and decreased fertility are more common in type 1 diabetes mellitus (DM1) patients who are at higher risk of hypoglycemia compared to those with type 2 diabetes [9]. Furthermore poor semen quality has also been reported in DM1 men, including decreased sperm motility and concentration, abnormal morphology, and increased seminal plasma abnormalities [10, 11]. In addition, diabetic men may have decreased serum testosterone due to impaired Leydig cell function in line with SCOS [12]. The reason for this was not conclusively investigated. Mallidis et al. reported autoimmunity to be the likely etiopathogenesis of gonadal dysfunction in DM1 [9]. Thus the insulinoma-primary hypogonadism syndrome needs to be identified so as not to misdiagnose autoimmune polyglandular failure with a combination of Addison's disease which may present with hypoglycemia and autoimmune testicular failure [13]. Our index case had no clinical and/or biochemical evidence of autoimmune related diseases.

## 4. Conclusion

We report a case of insulinoma presenting with neuroglycopenic symptoms, primary testicular failure, and Sertoli cell only syndrome. The likely link had been discussed. Considering that this is a single case report, further prospective study on a larger population at risk of recurrent hypoglycemia and infertility is needed to confirm our findings.

## Figures and Tables

**Table 1:** Serum hormone concentrations and semen parameters in patient with insulinoma.

	Ref range	Result
Test		
Follicle stimulating hormone (mIU/L)	<10	28
Luteinizing hormone (mIU/L)	<9	8
Testosterone (ng/dL)	300–1200	291
Free testosterone (ng/dL)	9–30	5.3
Sex hormone binding globulin (nmo/L)	10.0–70.0	38
Estradiol (pg/mL)	10–40	25
Prolactin (mIU/L)	<500	543
Seminal fluid analysis		
Age at testing (hr·min)	<2.01	1.05
Semen volume (mL)	>1.4	2
Viscosity	—	↑↑
Sperm concentration	>14	0
Leucocytes	<1	<1
